# Path to Suicidality in Korean Adolescents: Mediating Role of Self-Esteem, Somatic Symptoms, and Self-Harm Amid Depressive Symptoms

**DOI:** 10.3390/healthcare12161662

**Published:** 2024-08-20

**Authors:** Jiyeon Kim, Myongsun Cho

**Affiliations:** 1 College of Nursing, Ewha Womans University, Seoul 03760, Republic of Korea; jiyeonk@ewha.ac.kr; 2 Department of Nursing, Gangneung-Wonju National University, Wonju 26403, Republic of Korea

**Keywords:** adolescent health, suicidality, self-harm, somatization, depression, self-esteem

## Abstract

This study explored the relationship between depressive symptoms and suicidality among community-dwelling adolescents aged 10–18 years, examining whether self-esteem, somatic symptoms, and self-harm mediate this relationship. Utilizing a pre-existing dataset from a nationwide adolescent mental health survey conducted in Korea in 2021, data were collected using several standardized self-administered instruments: the Korean version of Rosenberg’s self-esteem scale, Korean Children’s Somatization Inventory, Korean version of the Self-Harm Inventory, Mental Health Screening for Depressive Disorders, and Mental Health Screening for Suicide Risk. A path model was constructed and validated, followed by path analysis to assess the effects. Data from 6689 adolescents, including 5937 students and 752 out-of-school adolescents, revealed that 18.7% were in the suicidality group, 11.8% experienced depressive symptoms, 57.9% exhibited somatic symptoms, and 27.4% engaged in self-harm. Depressive symptoms had a positive direct effect on suicidality (β = 0.166, *p* < 0.001, 95% confidence interval = 0.159–0.172). Bootstrapping tests showed a statistically significant indirect effect of self-esteem, somatic symptoms, and self-harm on the relationship between depressive symptoms and suicidality (β = 0.021, 95% confidence interval = 0.013–0.029). Our findings suggest that self-esteem, somatic symptoms, and self-harm mediate the relationship between depressive symptoms and suicidality, and comprehensive mental health management strategies addressing these factors are recommended.

## 1. Introduction

Suicide is a leading cause of death among adolescents worldwide. According to the World Health Organization (WHO) [1], suicide was the fourth leading cause of death among adolescents and young adults aged 15–29 years in 2019, regardless of gender. In 2020, Korea’s suicide rate was 24.1 per 100,000 people; it has witnessed the highest suicide rates among the Organization for Economic Co-operation and Development member countries since 2003 [2]. According to the 2022 Children and Youth Well-being in Korea report [3], the suicide rate among 15–17-year-olds was 9.5 per 100,000 people; among 12–14-year-olds, it increased sharply from 1.3 in 2016 to 3.1 in 2018 and 5.0 in 2021. In addition to the estimations of suicide rates, the percentage of adolescents reporting suicidal ideation or attempting suicide rose to 12.7% and 2.2% in 2021, respectively, up from 10.9% and 2.0% in 2020. When considering causes of death, “intentional self-harm (suicide)” accounted for the largest proportion (43.7%) of the causes of death of adolescents aged 10–19 years [4].

In 2018, the National Human Rights Commission reported that mental illness could worsen during teenage years because of factors such as parents’ lack of awareness of mental health problems, social prejudice and stigma, and limited access to early screening and treatment owing to a lack of appropriate health service infrastructure [5]. The Ministry of Health and Welfare proposed policies that included a survey of mental illness, revision of related laws, and expansion of treatment infrastructure [6]. Therefore, intentional self-harm and suicide among adolescents are considered significant public health problems.

Depressive symptoms are a significant predictor of suicide in adolescents [7]. When considering suicide motives among adolescents aged 11–20 years, in 2021, the rate of suicide due to mental and psychiatric problems was 58.6%, the highest among suicidal motivations [8]. Korean adolescents sometimes experience excessive stress related to academic and career concerns, and this stress can cause depression. The rate of stress perception and experience of depression among adolescents aged 9–24 years in 2021 were 38.8% and 26.8%, respectively [9]. Depressive disorder in adulthood can be a consequence of depression during adolescence, necessitating timely and appropriate management [10]. Depressive symptoms have a strong association with suicidality in both adolescents [11] and young adults [12]. Korean adolescents tend to be less likely to disclose depression owing to the social stigma associated with seeking help for mental health problems [13,14]. Consequently, screening for suicide risk may pose challenges, particularly for community adolescents with depression who have not been diagnosed with symptoms [15].

The presence of severe depressive symptoms can be predicted in adolescents who report somatic symptoms [13]. Evidence indicates a positive association between the severity of depression and somatic symptoms [16,17]. Adolescents with moderate to severe depressive symptoms experience growth problems and physical disability symptoms more commonly compared to adults [14]. Somatic symptoms appear as a physical reaction when one denies or minimizes depressive symptoms; they include pain, fatigue, weakness, dyspnea, dizziness, palpitations, gastrointestinal complaints, paresthesia, and tinnitus [18]. In Korea, 10–40% of patients who clinically complain of somatic symptoms have an unknown cause, and 11.5% of patients visiting general hospitals for the first time in departments other than psychiatry have somatic symptoms [19]. Despite this, somatic symptoms are not viewed as major issues for the mental health of Korean teenagers.

Self-harm, defined as the intentional infliction of harm on oneself, serves as a significant risk factor for repeated self-harm, suicide attempts, and potentially subsequent death [20,21,22]. Self-injurious behavior begins in the pubertal phase of adolescence, marked by vulnerability to negative social cues; among community adolescents, self-cutting is the most common behavior noted as non-suicidal self-injury [23]. Further, adolescents with higher levels of depression are more likely to engage in self-harm [21,24]. During the emotionally vulnerable period of adolescence, psychological problems such as depression increase the risk of self-destructive behavior throughout life [23] and act as a risk factor for suicide attempts [25].

Self-esteem refers to an individual’s evaluation of their own value and importance [26], and has an impact on the degree of psychological vulnerability one experiences [27]. During adolescence, self-esteem is a crucial psychological resource for developing self-identity. Additionally, self-esteem helps adolescents develop the internal capacity to overcome crises that threaten their mental health [6]. Adolescents’ self-esteem is a significant protective factor against mental health issues such as depression and suicidality [23,27,28,29,30].

According to the American Psychological Association, suicidality encompasses the potential for suicide, typically signaled by either suicidal thoughts or intentions, particularly when accompanied by a detailed suicidal plan; this definition may also encompass suicidal ideation, plans, gestures, or actual attempts [31]. With the number of suicides among Korean teenagers increasing, the number of self-harm cases has also been increasing. According to 2018–2022 data from the Central Emergency Medical Center’s National Emergency Medical Information Network, the number of emergency room visits due to self-harm and suicide attempts among teenagers also increased by 52.5%, from 4944 in 2018 to 7540 in 2022 [32]. Among the most frequent disease codes for teenagers visiting emergency rooms due to self-harm and suicide attempts in 2022, “depressive episode” ranked second [32], indicating a close association between depression and suicide attempts. Although depression, self-injurious behavior with relatively low medical severity, and somatic symptoms of unknown causes are potential risk factors for adolescent suicide, they are often overlooked as seeking help is constrained by a lack of understanding and fear of stigma. For early detection and intervention concerning suicide risk factors, the WHO’s Mental Health Gap Action Programme (mhGAP) guidelines [33] recommend initial and regular evaluations of mental status (depression and psycho-behavioral disorders), medically serious acts of self-harm, and somatic symptoms. Morales-Chainé et al. [16] examined the multifaceted relationship between depression, self-harm thoughts and behavior, somatic symptoms, and suicidal behavior among adults above 18 years old during the COVID-19 pandemic. Zou et al. [30] also reported that core self-evaluation plays a significant mediating role between depressive symptoms and suicide ideation in adolescents. Studies have also reported that adolescents with high self-esteem have a significantly lower probability of committing suicidal behavior compared to adolescents with low self-esteem [11,29].

However, few studies have tested the mediating effects of somatic symptoms, self-harm, and self-esteem in the relationship between depression and suicidality in community-based adolescents. Although previous studies have explained depression [11,25,34], self-harming behavior [25,35], somatic symptoms [13,25], or self-esteem [28,30,36] as predictors of adolescent suicidality, it is difficult to understand the relationship between these variables clearly. Therefore, this study aimed to explore the mediating effects of self-harm, somatic symptoms, and self-esteem in the relationship between depressive symptoms and suicidality using survey data from community-dwelling adolescents aged 10–18 years in Korea. In this regard, the following hypotheses were proposed:

**H1:**  *Depressive symptoms are associated with suicidality*.

**H2:**  *Self-esteem mediates the relationship between depressive symptoms and suicidality*.

**H3:**  *Somatic symptoms mediate the relationship between depressive symptoms and suicidality*.

**H4:**  *Self-harm mediates the relationship between depressive symptoms and suicidality*.

**H5:**  *Self-esteem, somatic symptoms, and self-harm play serial mediating roles in the relationship between depressive symptoms and suicidality*.

## 2. Materials and Methods

We used raw data from the 2021 National Survey on the Mental Health Status of Adolescents, involving adolescents aged 10–18 years, conducted in 2021 by the National Institute of Youth.

The survey employed stratified cluster sampling to sample youth who were enrolled in school, encompassing students from the fourth grade of elementary school to the third grade of high school based on data from the 2020 Education Statistics Yearbook [6]. The school was the unit of sampling in the first stage. A sample class was selected from the extracted school and all students in the class were surveyed until the overall target sample size (elementary school: 2000; middle school: 2000; high school: 2000) was reached. Post-stratification weighting was applied for gender. For out-of-school youth, a quota sampling method was used to allocate a total sample of 700 adolescents to five regions using the 2021 Dream Center list. Statistics on center use by out-of-school youth in 2020, used as a sampling frame, have a fundamental limitation in that the population cannot be estimated stably as it may vary from year to year [6]. The survey was conducted with a final sample comprising 5937 in-school youth and 752 out-of-school youth, resulting in 6689 adolescents aged 10–18 years who agreed to participate in 2021. Based on the school level, there were 1997 participants in the 4th–6th grades of elementary school, 1959 in the 1st–3rd grades of middle school, and 1982 high school students [6]. The study was conducted according to the guidelines of the Declaration of Helsinki and approved by the Institutional Review Board of the National Youth Policy Institute (202106-HR-GOYU-009, June 2021). Before participating in the survey, study information letters were distributed to adolescents through elementary, middle, and high schools nationwide, as well as through out-of-school institutions. Adolescents who voluntarily expressed their willingness to participate provided informed consent before their data were collected via web or mobile surveys using codes provided by their teachers.

Data collection occurred between July and August 2021, through a self-administered online survey (web or mobile) targeting teenagers. The purpose was to assess the mental health status of adolescents and to generate foundational data for developing policies that could support improving adolescent mental health.

The survey items covered three main areas: (1) clinical assessments for issues such as depression, anxiety, suicide, self-harm, stress, attention deficit hyperactivity disorder, and somatization; (2) exposure to protective and risk factors that contribute to major mental health problems; and (3) adolescents’ awareness, experience, and perception of various youth mental health promotion services [6].

### 2.1. Research Framework

**Figure 1:**
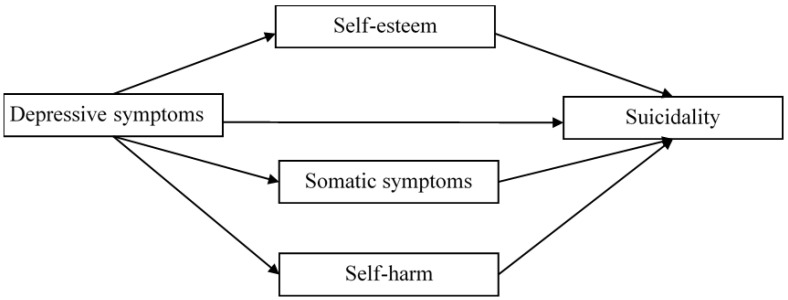
Research framework showing the relationships between depressive symptoms, self-esteem, somatic symptoms, self-harm, and suicidality with the mediation model.

Figure 1 presents the factors affecting suicidality examined in this study, based on the WHO’s mhGAP framework [33] and key risk factors associated with adolescent suicidality presented by Hawton et al. [37]. Suicidality is the final product of the dynamic interrelationship between depressive symptoms, self-esteem, somatic symptoms, and self-harm. Suicidality is also influenced by various background and contextual variables such as gender, school level, school attendance status, type of family, type of living, household economic status, and stress.

### 2.2. Measurements

#### 2.2.1. Dependent Variable

Suicidality was measured using the Mental Health Screening for Suicide Risk (MHS:S). MHS:S was introduced following comprehensive validation, which confirmed its reliability and validity in samples including Korean adolescents [6,38]. MHS:S consists of four questions on suicidal thoughts and plans over the past two weeks and past suicide attempts. Each item is rated on a 5-point Likert scale (0 = never true, 1 = slightly true, 2 = true, 3 = mostly true, 4 = completely true). The Cronbach’s alpha was 0.862 for the sample in this study.

#### 2.2.2. Independent Variable

Mental Health Screening for Depressive Disorders (MHS:D) was used to assess depression. It comprises 12 items, including nine core diagnostic criteria: depressed mood, decreased interest, changes in appetite or weight, changes in sleep, psychomotor agitation or retardation, fatigue, feelings of worthlessness or guilt, difficulty concentrating or indecisiveness, and suicidal thoughts and attempts. The items are rated on a 5-point Likert scale to report the extent to which the symptoms were experienced during the past two weeks (0 = never, 1 = slightly, 2 = yes, 3 = quite a bit, 4 = extremely). High reliability and validity in a sample of Korean adolescents have been previously reported [39]. The Cronbach’s alpha was 0.913 for the sample in this study. 

#### 2.2.3. Mediating Variables

The Korean Children’s Somatization Inventory was used to evaluate somatic symptoms. It comprises 36 items, and the degree to which it is difficult to treat each physical symptom is evaluated on a 4-point Likert scale (0 = no symptoms, 1 = slightly difficult, 2 = moderately difficult, 3 = severely difficult). A validation study with Korean adolescents reported an internal consistency reliability of 0.87 and a test-retest reliability of 0.88, indicating a satisfactory level of reliability [40]. The Cronbach’s alpha was 0.871 for the sample in this study.

The Korean version of the Self-Harm Inventory (K-SHI) [41] was used to evaluate self-harm symptoms. The scale comprises 22 items evaluated on a 2-point scale (1 = yes, 0 = no). The K-SHI has shown good reliability (test-retest reliability: 0.75; internal consistency reliability: 0.76), in addition to confirming acceptable convergent and discriminant validity [41]. In the 2021 National Survey on the Mental Health Status of Adolescents, the Institutional Review Board identified concerns that some of the 22 original items could potentially infringe upon the rights of Korean adolescents. Consequently, self-harm symptoms were assessed using a modified set of five items: (1) Did you intentionally cut yourself with a knife? (2) Did you hit yourself? (3) Did you purposefully bang your head? (4) Did you intentionally scratch your body to the point of pain? (5) Did you deliberately engage in actions to cause injury? [6]. The Cronbach’s alpha was 0.877 for the sample in this study.

The Korean version of Rosenberg’s [26] self-esteem scale was used to measure self-esteem. The scale consists of 10 items measured on a 4-point Likert scale (1 = not at all true, 2 = somewhat true, 3 = somewhat true, 4 = very true). The Cronbach’s alpha was 0.859 for the sample in this study.

#### 2.2.4. Control Variables

Based on a literature review [6,37], sociodemographic and psychological variables identified as affecting suicidality were selected as covariates. Sociodemographic variables included gender, school attendance status, household economic status, type of family, and type of living. Psychological variables included stress.

Gender was classified as male and female. School attendance status was categorized into school youth and out-of-school youth. School level, a proxy variable for age, was categorized into “elementary school”, “middle school”, and “high school” for school youth, and “below middle school (10–15 years)” and “high school (16–18 years)” for out-of-school youth. Type of family was classified as “family with diverse cultural backgrounds” and “family with Korean cultural backgrounds”. Type of living was categorized into “living with family” and “dormitory/boarding/living alone”.

The Korean Version of the Perceived Stress Scale for Adolescents (KPSS-A) was used to measure stress. This scale consists of 10 items evaluated on a 5-point scale (0 = never, 1 = rarely, 2 = sometimes, 3 = quite often, 4 = very often). In a validation study targeting Korean adolescents, the KPSS-A had an acceptable internal reliability of 0.77 and a test-retest reliability of 0.82 [42]. Additionally, convergent and discriminant validity were confirmed, along with its suitability for adolescent samples [42]. The Cronbach’s alpha was 0.832 for the sample in this study.

### 2.3. Statistical Analysis

The chi-square test was used to assess differences between the suicidality and no-suicidality groups. We examined the factors affecting suicidality using two regression models. Model 1 included the variables school level, gender, school attendance status, type of family, type of living, and stress. In Model 2, depression, self-esteem, somatic symptoms, and self-harm were added to the variables in Model 1.

Statistical significance was defined as a *p*-value < 0.05, and adjusted *p*-values with beta coefficient was calculated using SPSS Statistics 27.0 (IBM Corp, Armonk, NY, USA). The PROCESS macro v4.2 program [Model 4] was used to analyze the mediating effect of self-esteem, somatic symptoms, and self-harm in the relationship between depressive symptoms and suicidality. In the analysis of mediation effects, we employed bootstrapping, a method that circumvents the incorrect assumption of normal distribution for mediation effect estimates, as suggested by Sobel [43]. This approach allows for a more accurate estimation of effects without relying on normal distributional assumptions [44].

## 3. Results

### 3.1. Results Based on Sample Characteristics

Table 1 presents the descriptive statistics for weighted dataset. The survey involved 6689 adolescents, with 50.0% male and 50.0% female participants. 18.7 percent of the total participants were classified into the group with suicidality, while 11.8% experienced depressive symptoms, 57.9% exhibited somatic symptoms, and 27.4% reported self-harm. Additionally, out-of-school youth represented 11.2%, leaving the remaining 88.8% as school students. Among the latter group, elementary school students comprised 33.6%, with both middle and high school students each accounting for 33.0% and 33.4%. The two groups, suicidality and no-suicidality, showed differences across several variables (Table 1). There was a higher proportion of female adolescents (61.3%), adolescents not living with their families (5.6%), and adolescents from multicultural families (5.0%) in the suicidality group than in the no-suicidality group. Further, the suicidality group had significantly higher stress levels, somatic symptoms, self-harm, and lower self-esteem and household economic status compared to the no-suicidality group.

### 3.2. Factors Affecting Suicidality

Table 2 shows the results of the linear regression analysis estimating the effects of the control variables (Model 1) and the effects of self-esteem, self-harm, somatic symptoms, and depressive symptoms on suicidality after controlling for the effects of gender, school attendance status, school level, type of family, type of living, household economic status, and stress (Model 2). The index for the variance inflation factor was 1.02–2.16; multicollinearity was not identified; the Durbin–Watson test value was 2.009; and there was no autocorrelation between independent variables. In Model 1, living with family (β = −0.039, *p* < 0.001) and household economic status (β = −0.058, *p* < 0.001) were found to be significant protective factors against suicidality, while being female (β = 0.025, *p* = 0.031), having a multicultural family background (β= 0.037, *p* = 0.001), and stress (β = 0.313, *p* < 0.001) were risk factors for suicidality. In Model 2, the newly added depressive symptoms (β = 0.604, *p* < 0.001), somatic symptoms (β = 0.031, *p* = 0.002), and self-harm (β = 0.133, *p* < 0.001) were found to be significant risk factors for suicidality, while self-esteem (β =−0.044, *p* < 0.001) was found to be a significant protective factor for suicidality. Additionally, being female (β = 0.020, *p* = 0.019), school level (β = −0.037, *p* < 0.001), being an out-of-school youth (β = 0.074, *p* < 0.001), living with family (β = −0.021, *p* = 0.012), and stress (β = 0.021, *p* = 0.029) were found to be significant factors for suicidality. However, among the covariates, household economic status and multicultural families, which were significant in Model 1, no longer showed significance in suicidality in Model 2. Model 1 explained approximately 15.1% of the variance in suicidality; this increased to 53.6% in Model 2 (Table 2). 

### 3.3. Mediation Model

**Figure 2:**
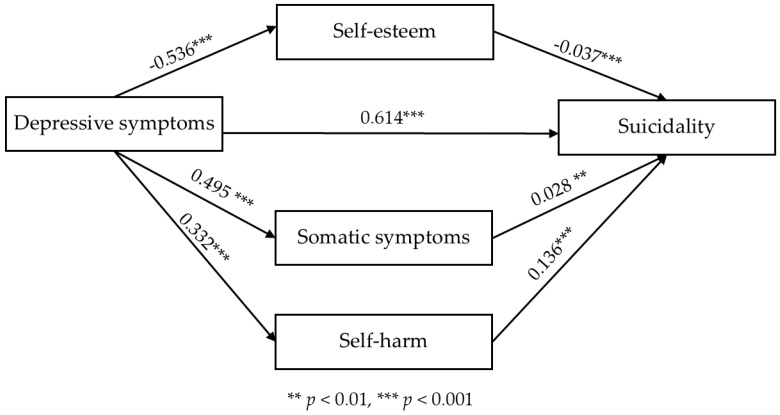
The serial mediation model of self-esteem, somatic symptoms, and self-harm in the relationship between depressive symptoms and suicidality among Korean adolescents.

Figure 2 illustrates the structural path model relationships among the key study variables. The model was saturated, with significant effects of all factors and all relationships in the anticipated directions. The path model results revealed the effects of depressive symptoms on suicidality through the mediation of self-esteem, somatic symptoms, and self-harm. The effect size is shown in Figure 2.

Table 3 presents the total effect and direct effect of depressive symptoms on suicidality. Depressive symptoms had a positive association with suicidality (β = 0.166, *p* < 0.001, 95% confidence interval = 0.159–0.172). The total effect size was 0.187 (confidence interval = 0.182–0.192). The bootstrapping test for the indirect effect of self-esteem, somatic symptoms, and self-harm on the relationship between depressive symptoms and suicidality showed a statistically significant association (β = 0.021, 95% confidence interval = 0.013–0.029). In other words, self-esteem, somatic symptoms, and self-harm partially mediated the relationship between depressive symptoms and suicidality, consistent with the study Hypothesis H5.

The simple mediating effects of self-esteem (β = 0.005, confidence interval = 0.002–0.009) and self-harm (β = 0.012, confidence interval = 0.010–0.015) were significant, whereas the simple mediating effect of somatic symptoms was not significant (β = 0.004, confidence interval = −0.002–0.010). Additionally, the path from depressive symptoms to self-harm revealed that suicidality increased as self-harm increased, and depressive symptoms were the strongest predictor of suicidality (Figure 2 and Table 3).

## 4. Discussion

The present study investigated the relationship between depressive symptoms and suicidality among community adolescents, focusing on the mediating roles of self-esteem, somatic symptoms, and self-harm, as well as the influence of various background and contextual variables. Regression analysis found significant effects of sociodemographic variables (school level, female, out-of-school youth, and type of living) and mental health variables (self-esteem, depressive symptoms, somatic symptoms, and self-harm) on suicidality. The model explained 53.6% of the variance in the dependent variable through the independent variables, indicating a relatively high explanatory power. The mediation effect test confirmed a partial mediating effect of self-esteem, somatic symptoms, and self-harm in the relationship, wherein depressive symptoms had a positive association with suicidality. The findings provide valuable insights into the mechanisms linking depressive symptoms to suicidality in this population.

In terms of sociodemographic variables confirmed through regression analysis, being female, school level, being an out-of-school youth, and types of living emerged as significant predictors of suicidality. Female adolescents had higher suicidality rates than males, consistent with prior studies [15,25,29,30,45]. Luby and Kertz [46] explained that increasing suicide rates in early adolescent girls (10–14 years) in the United States are associated with the rise of social media use. Unrestricted social contact through social media leads to interpersonal stress or cyberbullying, which is a significant risk factor for suicidality among female adolescents [46]. In Korea as well, the increasing use of social media may impact the mental health of female adolescents.

A significant difference in suicidality was found between below-middle school and high school age groups. There was a trend towards higher suicidality in the former, aligning with previous research [30,47]. Below-middle school age groups are at an increased risk of experiencing emotional problems owing to particular neurodevelopmental vulnerabilities that occur in the early stages of puberty [37]. Accordingly, the suicidality of middle-aged and younger adolescents may be higher compared to high school adolescents who are in the late or completed stages of puberty. 

Out-of-school youth exhibited significantly higher suicidality than school youth, echoing Wang et al.’s results [48]. Feeling connected to people has been reported as a protective factor for adolescent suicidality [45]. However, out-of-school youth face limitations in receiving protection from a stable social network system. The rate of Korean out-of-school youth experiencing symptoms of depression and anxiety was found to be three to four times higher than that of school youth, and the rate of experiencing delinquency was approximately six times higher [6]. Nonetheless, the main reasons for out-of-school youth not using mental health centers are the cost burden and fear of stigma associated with using mental health care, as reported in [6].

Adolescents living with their families had significantly lower suicidality, supporting the literature suggesting higher suicide risk among those not living with families [47,49]. Our results underscore the significant influence of family-related factors on adolescent mental health and suicidality. Adolescents experiencing parental loss or insufficient family attachment, coupled with feelings of depression and low self-esteem, are more likely to engage in self-harm as a means of coping with suppressed emotions such as anger and anxiety [50]. This behavior often serves as a maladaptive mechanism to manage intense emotional distress, ultimately increasing the risk of suicidality.

The hypotheses of our study were partially accepted through mediation effect analysis. Depressive symptoms had a significant direct effect on suicidality (H1), mediated by self-esteem (H2) and self-harm (H4). In addition, self-esteem, somatic symptoms, and self-harm were confirmed to play serial mediating roles in the relationship between depressive symptoms and suicidality (H5).

The findings of this study, which confirmed that depressive symptoms have a significant positive correlation with suicide, are consistent with previous studies [7,51]. Depressive symptoms in adolescence can lead to psychiatric disorders and increase the risk of self-destructive behavior across the lifespan, which is a factor in increasing suicidality [37]. In particular, it has been shown that mental problems are the main cause of suicide among Korean adolescents. Hence, adolescents with depressive symptoms need to be managed as a risk group for suicide [8].

Self-esteem had a partial mediating effect on the relationship between depressive symptoms and suicidality. Self-esteem has been reported in most previous studies to be a protective factor against suicidality [23,28,29,30]. Zou et al. [30] also reported that core self-evaluation has a mediating effect on the relationship between depressive symptoms and suicidal ideation in adolescents, aligning with our results. Therefore, enhancing self-esteem should be considered a major factor in preventing suicide among adolescents with depressive symptoms.

Self-harm had a partial mediating effect on the relationship between depressive symptoms and suicidality. These findings are consistent with Morales-Chainé et al.’s [16] path model, which confirmed the relationship between depression, self-harm, and suicidality. Self-harm is mainly caused by psychological factors, such as depressive symptoms, and is a significant predictor of suicide attempts [29]. Increased self-harm behaviors among adolescents were associated with higher suicidality levels [16,20,35]. Screening for non-suicidal self-injury among community youth can be difficult for medical professionals, often detected only after hospital admission owing to a medically serious suicide attempt. Therefore, the current results suggest that early detection and intervention not only for depression but also for self-harm behaviors are necessary to prevent suicide in adolescents.

Our study found that somatic symptoms did not have a significant simple mediating effect (indirect effect) on the relationship between depressive symptoms and suicidality. However, somatic symptoms showed a significant positive correlation with both depressive symptoms and suicidality, aligning with previous research [25,34]. Adolescents experiencing somatic symptoms often face additional stress and anxiety, which can exacerbate depressive symptoms and increase vulnerability to suicidal thoughts and behaviors. Addressing these physical manifestations of distress is crucial in comprehensive mental health interventions.

The path model suggested in our study confirmed the effect of depressive symptoms on suicidality through self-esteem, somatic symptoms, and self-harm. These results support Morales-Chainé et al.’s [16] findings, which show that previous self-harm thoughts and behaviors, marked distress, and somatic symptoms have mediating effects on the relationship between depressive symptoms and current self-harm or suicidal thoughts and behaviors in adults. Our findings indicate that low self-esteem, somatic symptoms, and self-harm may be significant risk factors for suicide in adolescents with depressive symptoms. Considering Korea’s cultural background, the social perception that suppressing feelings of discomfort is a virtue or the fear of being labeled a “mentally ill person” when talking about one’s experiences regarding depression may lead to reluctance to seek help, aggravating somatic symptoms, low self-esteem, or self-harm [13,16,37]. Accordingly, it is important to use a comprehensive tool that can accurately screen for depression or suicide risks that are not apparent in adolescents [15].

Given the established connections between school attendance, gender, family dynamics, depressive symptoms, self-esteem, somatic symptoms, self-harm, and suicidality, intervention strategies must be multifaceted and holistic. One effective approach is Dialectical Behavior Therapy (DBT), a modified form of Cognitive Behavioral Therapy (CBT) designed to treat individuals exhibiting self-harming behaviors, suicidal ideation, and suicide attempts [52]. DBT focuses on changing self-destructive behaviors and promoting the pursuit of a more fulfilling life. It helps individuals develop skills to manage distress, regulate emotions, and improve interpersonal relationships.

In the context of South Korea, the Ministry of Health and Welfare plays a pivotal role in enhancing public awareness of the value of life and providing medical-centered screening and management for high-risk groups such as individuals with severe mental illnesses and suicide survivors. Concurrently, the Ministry of Gender Equality and Family focuses on initiatives such as training gatekeepers in local communities, deploying peer supporters in adolescent support groups, and offering counseling services for adolescents. This study’s findings underscore the necessity of comprehensive intervention strategies that incorporate mental health support, behavioral monitoring, and the creation of nurturing environments to effectively mitigate the risk of suicidality in adolescents. Identifying at-risk individuals and providing timely, targeted support are critical measures in addressing the underlying factors contributing to suicidality. Schools, peer groups, and families need to play an effective role as “gatekeepers”. It is necessary to strengthen gatekeeper support systems centered on parents, peer groups, and schools to provide comprehensive awareness and prompt responses to depression, low self-esteem, somatic symptoms, and self-harm. Additionally, policy support is needed to reduce the taboo associated with suicide and mental health issues so that youth can talk about their difficulties and ask for help. Another important point concerns out-of-school youth. While the Ministry of Education’s youth suicide prevention activities are focused on school students, our results indicate higher suicidality among out-of-school youth, pointing to the need to strengthen accessibility to mental health services for this group.

### Limitations

This study has the following limitations: First, while the sample of school adolescents is highly representative, obtained using a stratified cluster sampling method, the sample of out-of-school adolescents relies on highly variable annual Dream Center usage statistics as a sampling frame, which may limit the generalizability of the results. Second, causal relationships between key variables cannot be confirmed due to the cross-sectional study design. Future research could benefit from a longitudinal study design to better establish causality. Lastly, the survey period may have influenced the results. Youth mental health-related variables could have been affected by the COVID-19 pandemic. Additionally, several studies have indicated that depressive symptoms are alleviated in summer compared to winter [53]. Since data for this study were collected during July and August, seasonal effects may have impacted the findings. Continuous evaluations are necessary to mitigate biases caused by seasonal changes or the pandemic.

## 5. Conclusions

Adolescent suicidality is influenced by a multifaceted interplay of factors, including mental health symptoms, behavioral issues, and contextual variables. Therefore, effective intervention strategies must adopt a holistic approach, addressing depressive and somatic symptoms, as well as self-harm behaviors, while also fostering protective environments both at home and in school. In addition, policy measures should be developed to strengthen out-of-school youth’s access to mental health and emotional support services. Early identification and targeted interventions for adolescents exhibiting depressive symptoms and self-harm behaviors are crucial in preventing suicidality and promoting mental well-being among adolescents in the Korean community. Future research should continue to explore these relationships longitudinally to better understand the causal pathways and to develop more effective prevention and intervention programs.

## Figures and Tables

**Table 1:** General characteristics of the no-suicidality and suicidality groups (*N* = 6689).

Factor		*N* (%) or M ± SD			t or χ^2^	*p*-Value
		Total (*N* = 6689)	No Suicidality (*N* = 5437)	Suicidality (*N* = 1252)		
Gender	Male	3343 (50.0)	2859 (52.6)	484 (38.7)	78.94	<0.001
	Female	3346 (50.0)	2578 (47.4)	768 (61.3)		
School level						
School youth	Elementary school	1997 (33.6)	1685 (34.0)	312 (32.0)	1.52	0.468
	Middle school	1959 (33.0)	1632 (32.9)	327 (33.5)		
	High school	1982 (33.4)	1645 (33.2)	337 (34.5)		
Out-of-school youth	Below middle school (10–15 years)	105 (14.0)	75 (15.8)	30 (10.8)	3.58	0.058
	High school (16–18 years)	647 (86.0)	400 (84.2)	247 (89.2)		
Type of family	Family with diverse cultural backgrounds	262 (3.9)	199 (3.7)	63 (5.0)	5.06	0.024
	Family with Korean cultural background	6428 (96.1)	5238 (96.3)	1190 (95.0)		
Type of living	Living with family	6431 (96.2)	5249 (96.6)	1182 (94.4)	12.74	<0.001
	Dormitory/Boarding/Living alone	257 (3.8)	187 (3.4)	70 (5.6)		
Household economic status (1–7)		4.57 ± 1.14	4.64 ± 1.11	4.26 ± 1.20	10.57	<0.001
Stress (0–40)		13.27 ± 7.36	12.09 ± 7.03	18.37 ± 6.50	−30.32	<0.001
Depressive symptoms (0–48)		4.92 ± 7.44	2.74 ± 3.95	14.42 ± 10.84	−37.58	<0.001
Self-esteem (0–40)		30.02 ± 5.95	31.33 ± 5.06	24.31 ± 6.15	37.60	<0.001
Somatic symptoms (0–108)		3.06 ± 5.59	2.09 ± 3.70	7.25 ± 9.27	−19.33	<0.001
Self-harm (0–5)		0.72 ± 1.43	0.49 ± 1.25	1.73 ± 1.72	−24.20	<0.001
Suicidality (0–16)		0.65 ± 2.00	0	3.49 ± 3.40	−36.39	<0.001

Note: M = mean and SD = standard deviation.

**Table 2:** Regression analysis of factors associated with suicidality (*N* = 6689).

	Model 1			Model 2		
Variable	β	t	*p*-Value	β	t	*p*-Value
Female	0.025	2.163	0.031	0.020	2.354	0.019
School level	0.004	0.314	0.753	−0.037	−4.004	<0.001
Out-of-school youth	0.166	13.543	<0.001	0.074	8.060	<0.001
Type of family	0.037	3.228	0.001	0.010	1.154	0.249
Type of living	−0.039	−3.448	<0.001	−0.021	−2.503	0.012
Household economic status	−0.058	−4.899	<0.001	0.014	1.550	0.121
Stress	0.313	27.342	<0.001	0.021	2.187	0.029
Self-esteem				−0.044	−4.271	<0.001
Depressive symptoms				0.604	49.348	<0.001
Somatic symptoms				0.031	3.069	0.002
Self-harm				0.133	14.761	<0.001
Adjusted R^2^	0.151			0.536		
F (*p*-value)	171.132 (*p* < 0.001)			699.508 (*p* < 0.001)		
Durbin–Watson	1.864			2.009		

**Table 3:** Direct and indirect effects of depressive symptoms on suicidality using 5000 bootstraps.

Path		Coeff	SE	*p*	LLCI	ULCI
Total effect	X→Y	0.187	0.003	<0.001	0.182	0.192
Direct effect	X→Y	0.166	0.004	<0.001	0.159	0.172
Indirect effect	Total	0.021	0.004	-	0.013	0.029
	X→M1→Y	0.005	0.002	-	0.002	0.009
	X→M2→Y	0.004	0.003	-	−0.002	0.010
	X→M3→Y	0.012	0.001	-	0.010	0.015

Note: X = depressive symptoms, M1 = Self-esteem, M2 = somatic symptoms, M3 = self-harm, and Y = Suicidality; Coeff: coefficient; SE: standard error; LLCI: lower level of the 95% confidence interval; ULCI: upper level of the 95% confidence interval.

## Data Availability

Data were made available by the National Youth Policy Institute after permission was obtained on 16 January 2024 (https://www.nypi.re.kr/archive/mps/program/examinDataCode/view?menuId=MENU00226&pageNum=2&titleId=160&schType=0&schText=&firstCategory=&secondCategory=, accessed on 16 January 2024).
